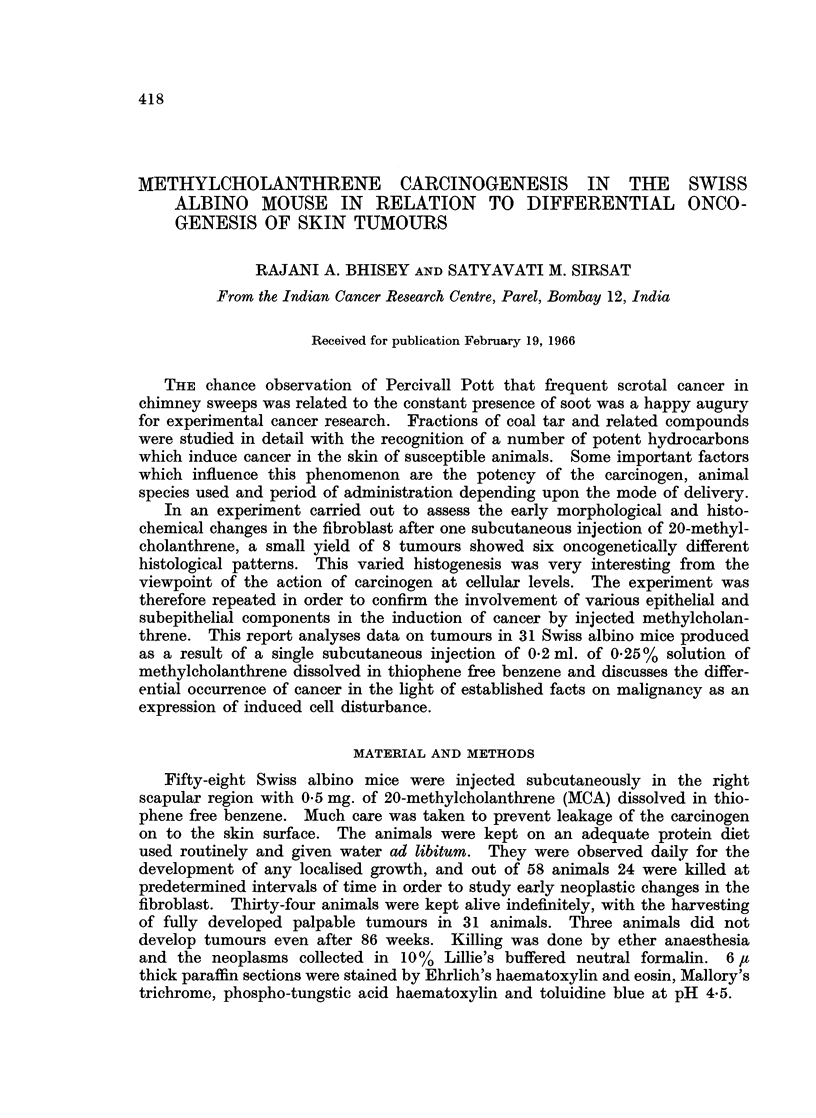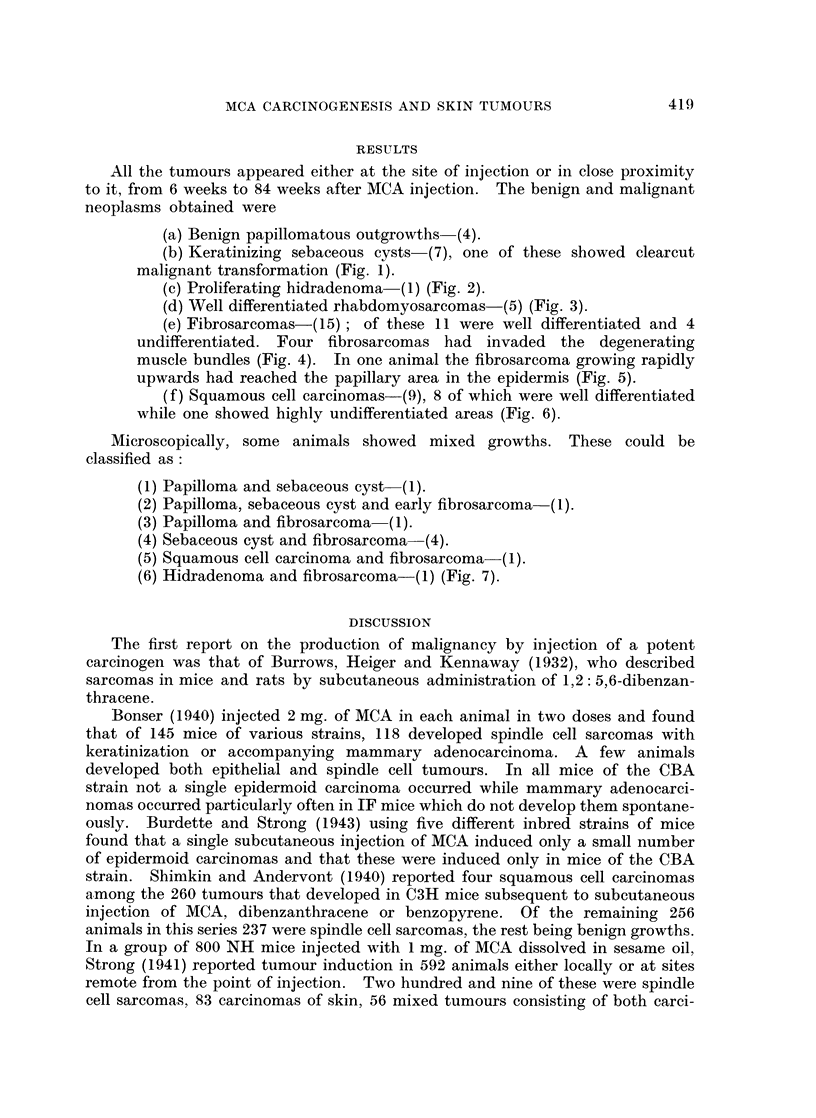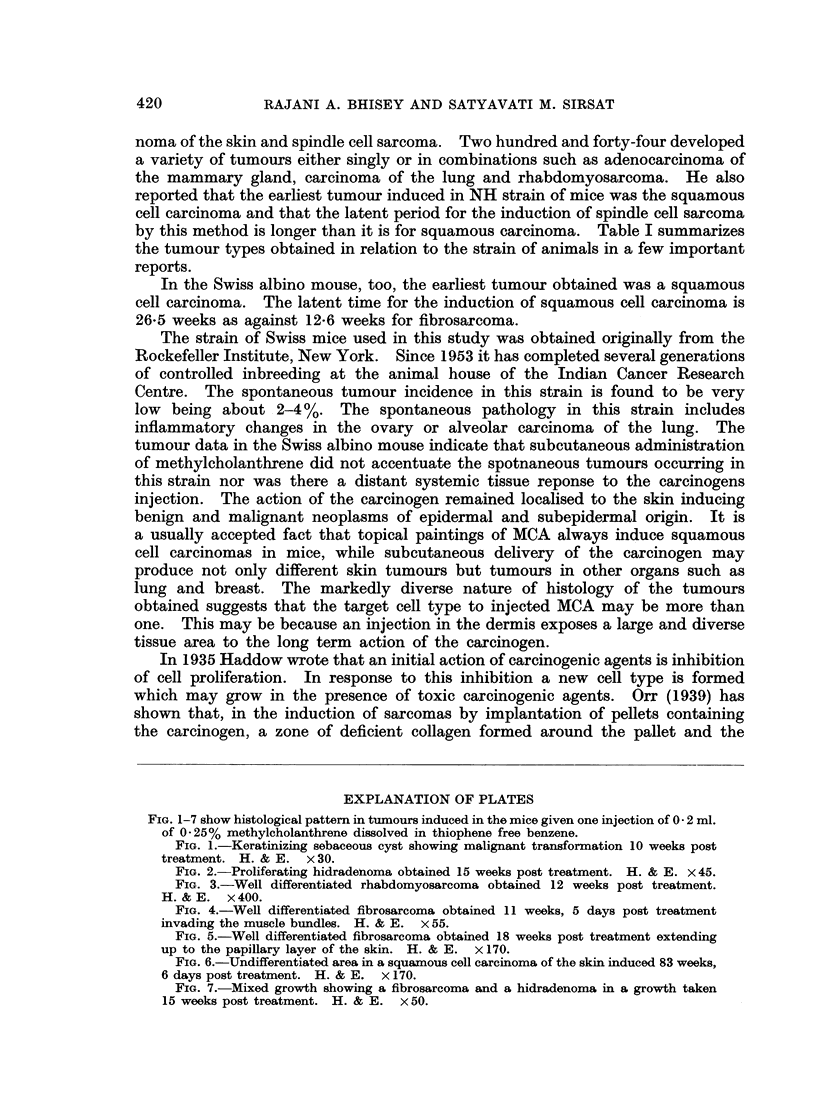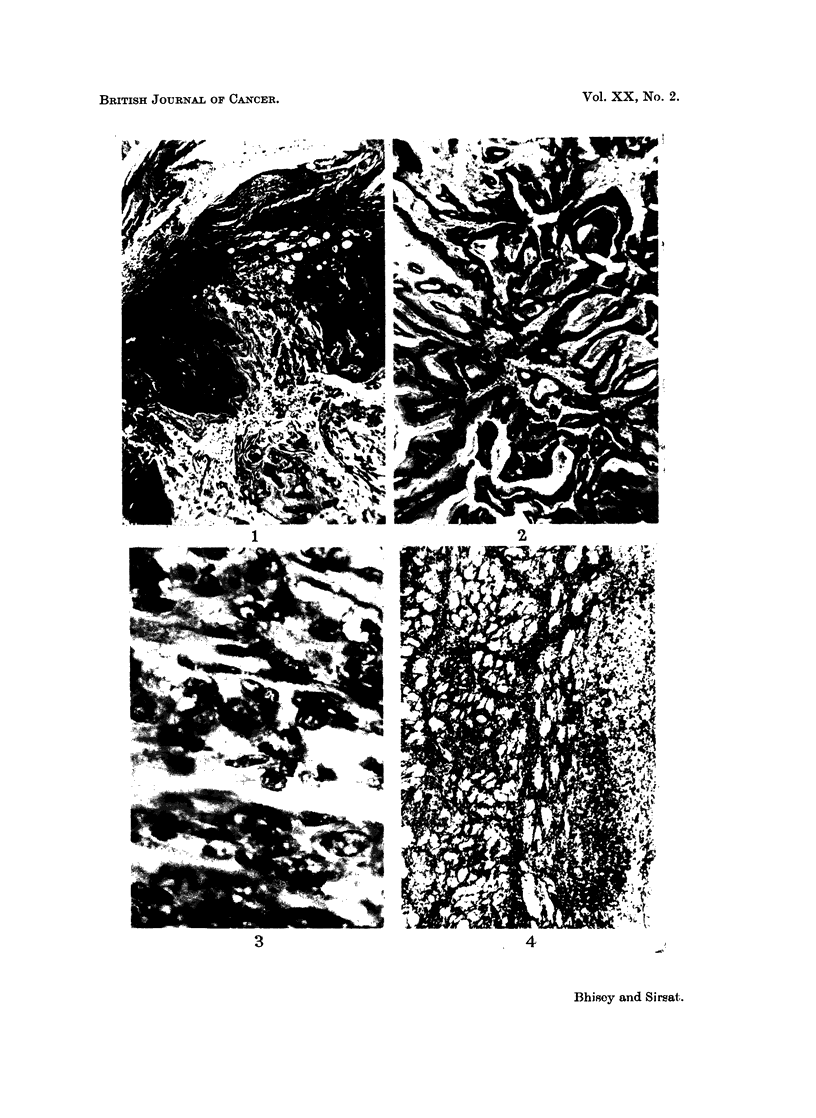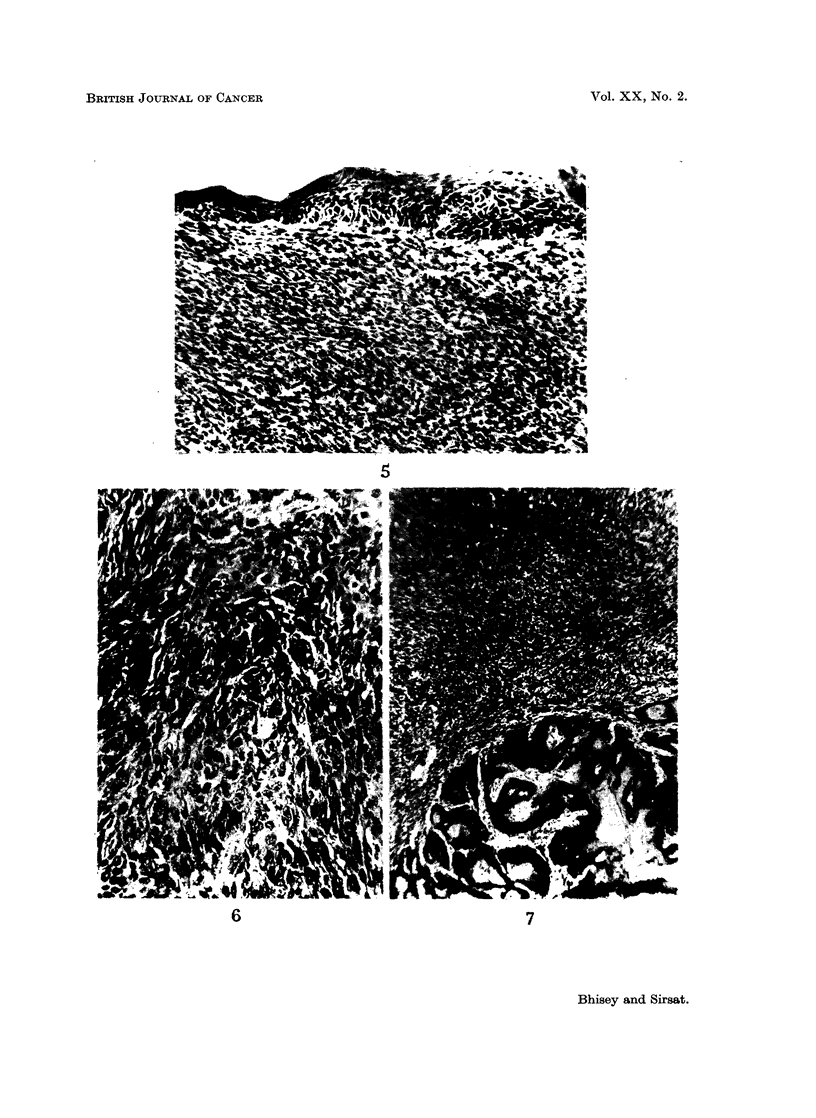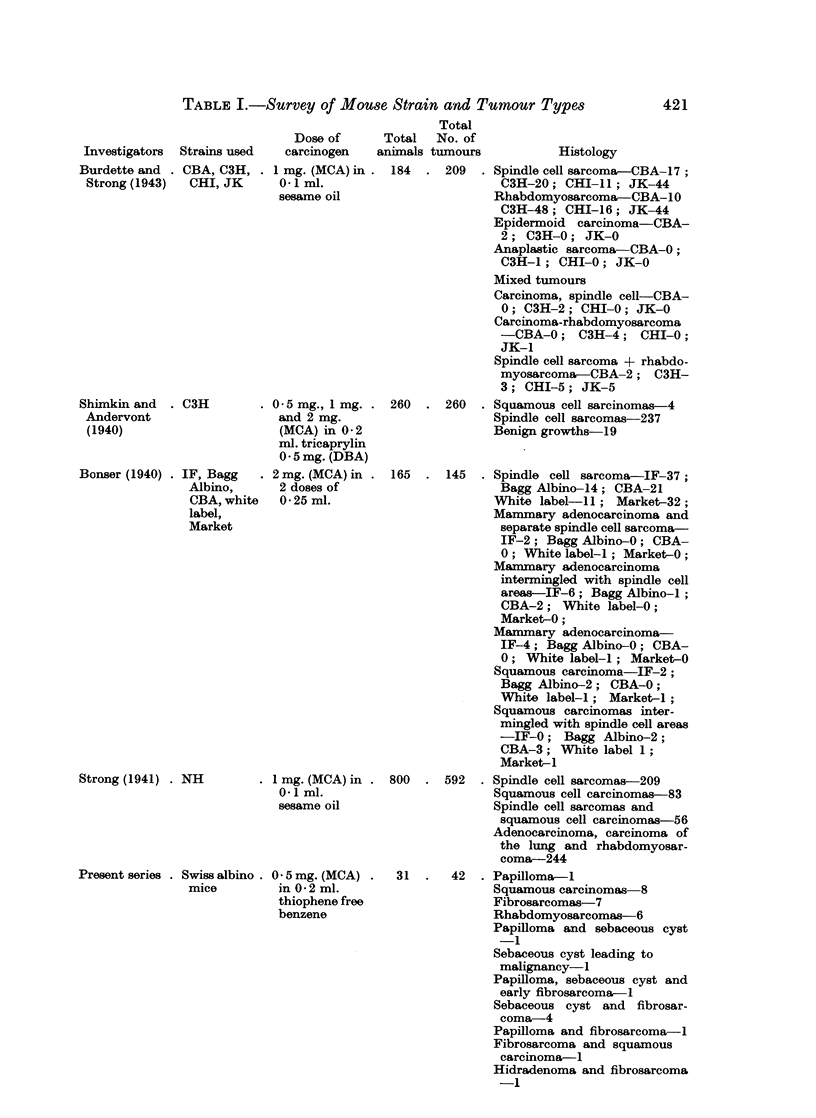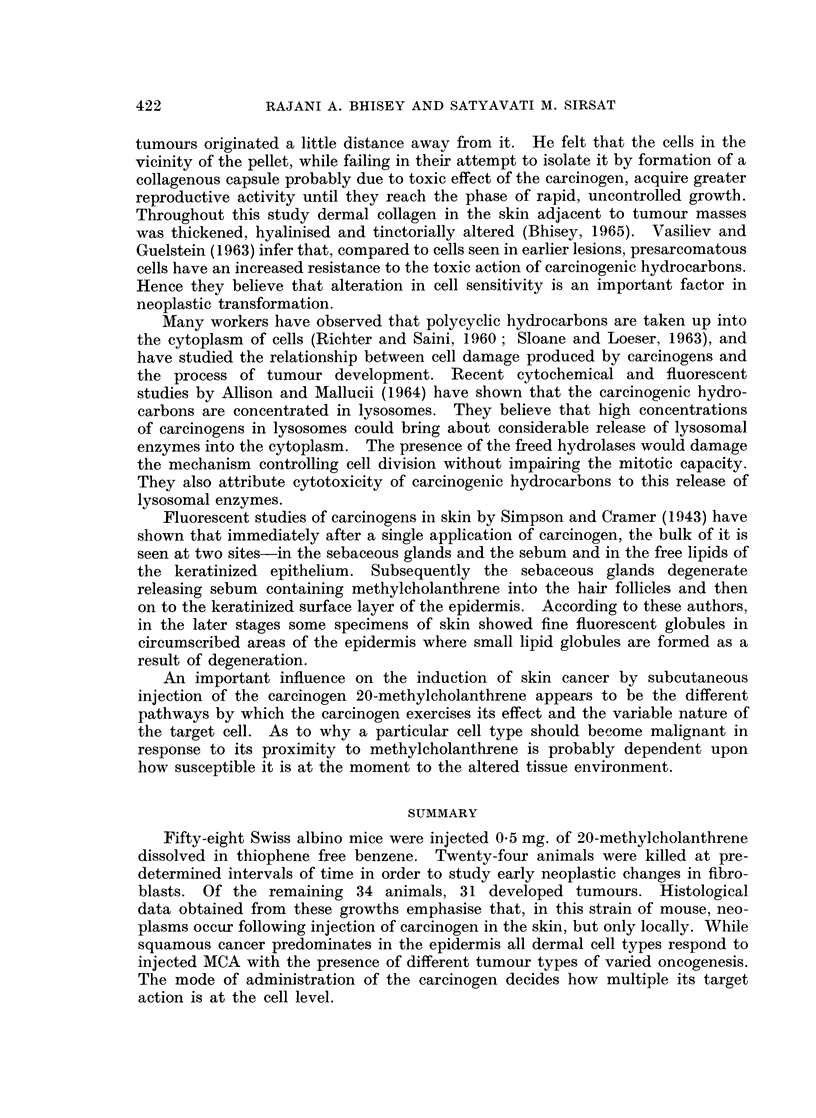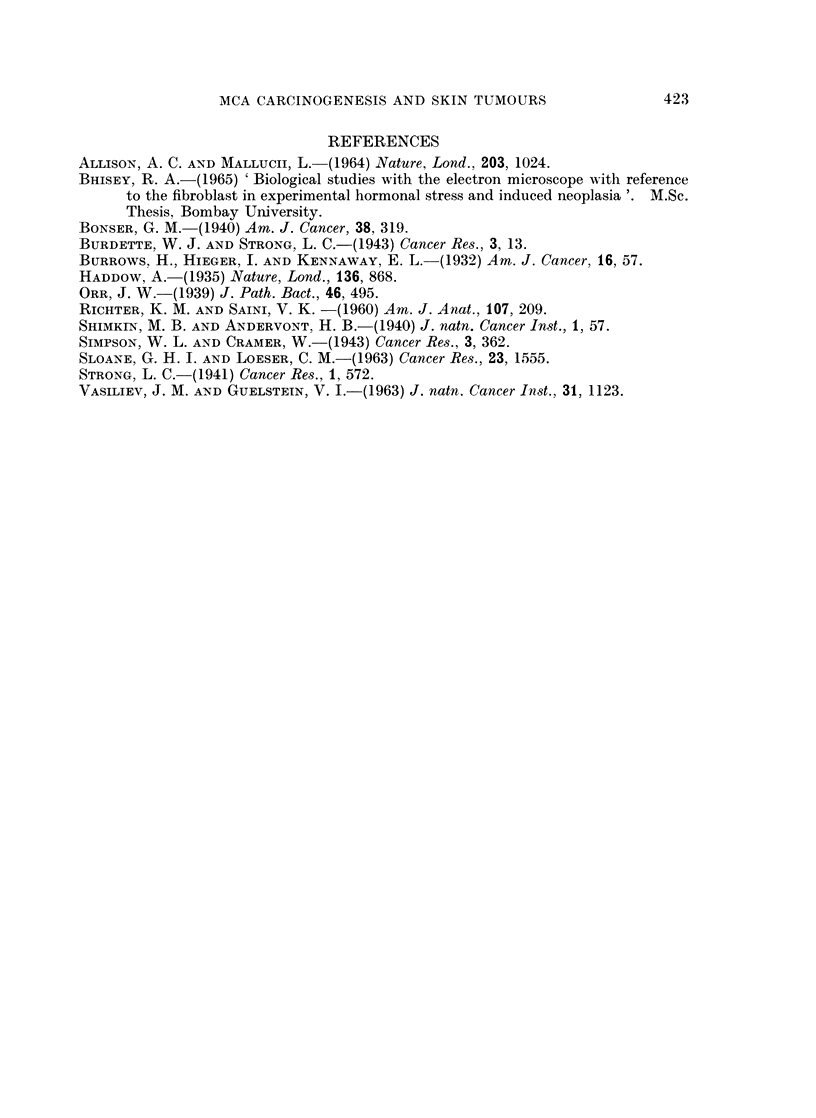# Methylcholanthrene carcinogens in the Swiss albino mouse in relation to differential oncogenesis of skin tumours.

**DOI:** 10.1038/bjc.1966.50

**Published:** 1966-06

**Authors:** R. A. Bhisey, S. M. Sirsat

## Abstract

**Images:**


### METHYLCHOLANTHRENE CARCINOGENESIS IN THE SWISS ALBINO MOUSE IN RELATION TO DIFFERENTIAL ONCOGENESIS OF SKIN TUMOURS

RAJANI A. BHISEY AND SATYAVATI M. SIRSAT

*From the Indian Cancer Research Centre, Parel, Bombay* 12, *India*

Received for publication February 19, 1966

THE chance observation of Percivall Pott that frequent scrotal cancer in chimney sweeps was related to the constant presence of soot was a happy augury for experimental cancer research. Fractions of coal tar and related compounds were studied in detail with the recognition of a number of potent hydrocarbons which induce cancer in the skin of susceptible animals. Some important factors which influence this phenomenon are the potency of the carcinogen, animal species used and period of administration depending upon the mode of delivery.

In an experiment carried out to assess the early morphological and histochemical changes in the fibroblast after one subcutaneous injection of 20-methylcholanthrene, a small yield of 8 tumours showed six oncogenetically different histological patterns. This varied histogenesis was very interesting from the viewpoint of the action of carcinogen at cellular levels. The experiment was therefore repeated in order to confirm the involvement of various epithelial and subepithelial components in the induction of cancer by injected methylcholanthrene. This report analyses data on tumours in 31 Swiss albino mice produced as a result of a single subcutaneous injection of 0·2 ml. of 0·25% solution of methylcholanthrene dissolved in thiophene free benzene and discusses the differential occurrence of cancer in the light of established facts on malignancy as an expression of induced cell disturbance.

### MATERIAL AND METHODS

Fifty-eight Swiss albino mice were injected subcutaneously in the right scapular region with 0·5 mg. of 20-methylcholanthrene (MCA) dissolved in thiophene free benzene. Much care was taken to prevent leakage of the carcinogen on to the skin surface. The animals were kept on an adequate protein diet used routinely and given water *ad libitum.* They were observed daily for the development of any localised growth, and out of 58 animals 24 were killed at predetermined intervals of time in order to study early neoplastic changes in the fibroblast. Thirty-four animals were kept alive indefinitely, with the harvesting of fully developed palpable tumours in 31 animals. Three animals did not develop tumours even after 86 weeks. Killing was done by ether anaesthesia and the neoplasms collected in 10% Lillie's buffered neutral formalin. 6 $\mu$ thick paraffin sections were stained by Ehrlich's haematoxylin and eosin, Mallory's trichrome, phospho-tungstic acid haematoxylin and toluidine blue at pH 4·5.

### RESULTS

All the tumours appeared either at the site of injection or in close proximity to it, from 6 weeks to 84 weeks after MCA injection. The benign and malignant neoplasms obtained were

(a) Benign papillomatous outgrowths—(4).

(b) Keratinizing sebaceous cysts—(7), one of these showed clearcut malignant transformation (Fig. 1).

(c) Proliferating hidradenoma—(1) (Fig. 2).

(d) Well differentiated rhabdomyosarcomas—(5) (Fig. 3).

(e) Fibrosarcomas—(15) ; of these 11 were well differentiated and 4 undifferentiated. Four fibrosarcomas had invaded the degenerating muscle bundles (Fig. 4). In one animal the fibrosarcoma growing rapidly upwards had reached the papillary area in the epidermis (Fig. 5).

(f) Squamous cell carcinomas—(9), 8 of which were well differentiated while one showed highly undifferentiated areas (Fig. 6).

Microscopically, some animals showed mixed growths. These could be classified as :

(1) Papilloma and sebaceous cyst—(1).
(2) Papilloma, sebaceous cyst and early fibrosarcoma—(1).
(3) Papilloma and fibrosarcoma—(1).
(4) Sebaceous cyst and fibrosarcoma—(4).
(5) Squamous cell carcinoma and fibrosarcoma—(1).
(6) Hidradenoma and fibrosarcoma—(1) (Fig. 7).

### DISCUSSION

The first report on the production of malignancy by injection of a potent carcinogen was that of Burrows, Heiger and Kennaway (1932), who described sarcomas in mice and rats by subcutaneous administration of 1,2 : 5,6-dibenzanthracene.

Bonser (1940) injected 2 mg. of MCA in each animal in two doses and found that of 145 mice of various strains, 118 developed spindle cell sarcomas with keratinization or accompanying mammary adenocarcinoma. A few animals developed both epithelial and spindle cell tumours. In all mice of the CBA strain not a single epidermoid carcinoma occurred while mammary adenocarcinomas occurred particularly often in IF mice which do not develop them spontaneously. Burdette and Strong (1943) using five different inbred strains of mice found that a single subcutaneous injection of MCA induced only a small number of epidermoid carcinomas and that these were induced only in mice of the CBA strain. Shimkin and Andervont (1940) reported four squamous cell carcinomas among the 260 tumours that developed in C3H mice subsequent to subcutaneous injection of MCA, dibenzanthracene or benzopyrene. Of the remaining 256 animals in this series 237 were spindle cell sarcomas, the rest being benign growths. In a group of 800 NH mice injected with 1 mg. of MCA dissolved in sesame oil, Strong (1941) reported tumour induction in 592 animals either locally or at sites remote from the point of injection. Two hundred and nine of these were spindle cell sarcomas, 83 carcinomas of skin, 56 mixed tumours consisting of both carcinoma of the skin and spindle cell sarcoma. Two hundred and forty-four developed a variety of tumours either singly or in combinations such as adenocarcinoma of the mammary gland, carcinoma of the lung and rhabdomyosarcoma. He also reported that the earliest tumour induced in NH strain of mice was the squamous cell carcinoma and that the latent period for the induction of spindle cell sarcoma by this method is longer than it is for squamous carcinoma. Table I summarizes the tumour types obtained in relation to the strain of animals in a few important reports.

In the Swiss albino mouse, too, the earliest tumour obtained was a squamous cell carcinoma. The latent time for the induction of squamous cell carcinoma is 26·5 weeks as against 12·6 weeks for fibrosarcoma.

The strain of Swiss mice used in this study was obtained originally from the Rockefeller Institute, New York. Since 1953 it has completed several generations of controlled inbreeding at the animal house of the Indian Cancer Research Centre. The spontaneous tumour incidence in this strain is found to be very low being about 2–4%. The spontaneous pathology in this strain includes inflammatory changes in the ovary or alveolar carcinoma of the lung. The tumour data in the Swiss albino mouse indicate that subcutaneous administration of methylcholanthrene did not accentuate the spotnaneous tumours occurring in this strain nor was there a distant systemic tissue reponse to the carcinogens injection. The action of the carcinogen remained localised to the skin inducing benign and malignant neoplasms of epidermal and subepidermal origin. It is a usually accepted fact that topical paintings of MCA always induce squamous cell carcinomas in mice, while subcutaneous delivery of the carcinogen may produce not only different skin tumours but tumours in other organs such as lung and breast. The markedly diverse nature of histology of the tumours obtained suggests that the target cell type to injected MCA may be more than one. This may be because an injection in the dermis exposes a large and diverse tissue area to the long term action of the carcinogen.

In 1935 Haddow wrote that an initial action of carcinogenic agents is inhibition of cell proliferation. In response to this inhibition a new cell type is formed which may grow in the presence of toxic carcinogenic agents. Orr (1939) has shown that, in the induction of sarcomas by implantation of pellets containing the carcinogen, a zone of deficient collagen formed around the pallet and the

---

EXPLANATION OF PLATES

FIG. 1–7 show histological pattern in tumours induced in the mice given one injection of 0·2 ml. of 0·25% methylcholanthrene dissolved in thiophene free benzene.

FIG. 1.—Keratinizing sebaceous cyst showing malignant transformation 10 weeks post treatment. H. & E. ×30.

FIG. 2.—Proliferating hidradenoma obtained 15 weeks post treatment. H. & E. ×45.

FIG. 3.—Well differentiated rhabdomyosarcoma obtained 12 weeks post treatment. H. & E. ×400.

FIG. 4.—Well differentiated fibrosarcoma obtained 11 weeks, 5 days post treatment invading the muscle bundles. H. & E. ×55.

FIG. 5.—Well differentiated fibrosarcoma obtained 18 weeks post treatment extending up to the papillary layer of the skin. H. & E. ×170.

FIG. 6.—Undifferentiated area in a squamous cell carcinoma of the skin induced 83 weeks, 6 days post treatment. H. & E. ×170.

FIG. 7.—Mixed growth showing a fibrosarcoma and a hidradenoma in a growth taken 15 weeks post treatment. H. & E. ×50.

1

2

3

4

Bhisey and Sirsat.

5

6

7

TABLE I.—*Survey of Mouse Strain and Tumour Types*

| Investigators | Strains used | Dose of carcinogen | Total animals | Total No. of tumours | Histology |
|---|---|---|---|---|---|
| Burdette and Strong (1943) | CBA, C3H, CHI, JK | 1 mg. (MCA) in 0·1 ml. sesame oil | 184 | 209 | Spindle cell sarcoma—CBA–17 ; C3H–20 ; CHI–11 ; JK–44<br>Rhabdomyosarcoma—CBA–10 C3H–48 ; CHI–16 ; JK–44<br>Epidermoid carcinoma—CBA–2 ; C3H–0 ; JK–0<br>Anaplastic sarcoma—CBA–0 ; C3H–1 ; CHI–0 ; JK–0<br>Mixed tumours<br>Carcinoma, spindle cell—CBA–0 ; C3H–2 ; CHI–0 ; JK–0<br>Carcinoma-rhabdomyosarcoma—CBA–0 ; C3H–4 ; CHI–0 ; JK–1<br>Spindle cell sarcoma + rhabdomyosarcoma—CBA–2 ; C3H–3 ; CHI–5 ; JK–5 |
| Shimkin and Andervont (1940) | C3H | 0·5 mg., 1 mg. and 2 mg. (MCA) in 0·2 ml. tricaprylin 0·5 mg. (DBA) | 260 | 260 | Squamous cell sarcinomas—4<br>Spindle cell sarcomas—237<br>Benign growths—19 |
| Bonser (1940) | IF, Bagg Albino, CBA, white label, Market | 2 mg. (MCA) in 2 doses of 0·25 ml. | 165 | 145 | Spindle cell sarcoma—IF–37 ; Bagg Albino–14 ; CBA–21 White label—11 ; Market–32 ;<br>Mammary adenocarcinoma and separate spindle cell sarcoma—IF–2 ; Bagg Albino–0 ; CBA–0 ; White label–1 ; Market–0 ;<br>Mammary adenocarcinoma intermingled with spindle cell areas—IF–6 ; Bagg Albino–1 ; CBA–2 ; White label–0 ; Market–0 ;<br>Mammary adenocarcinoma—IF–4 ; Bagg Albino–0 ; CBA–0 ; White label–1 ; Market–0<br>Squamous carcinoma—IF–2 ; Bagg Albino–2 ; CBA–0 ; White label–1 ; Market–1 ;<br>Squamous carcinomas intermingled with spindle cell areas—IF–0 ; Bagg Albino–2 ; CBA–3 ; White label 1 ; Market–1 |
| Strong (1941) | NH | 1 mg. (MCA) in 0·1 ml. sesame oil | 800 | 592 | Spindle cell sarcomas—209<br>Squamous cell carcinomas—83<br>Spindle cell sarcomas and squamous cell carcinomas—56<br>Adenocarcinoma, carcinoma of the lung and rhabdomyosarcoma—244 |
| Present series | Swiss albino mice | 0·5 mg. (MCA) in 0·2 ml. thiophene free benzene | 31 | 42 | Papilloma—1<br>Squamous carcinomas—8<br>Fibrosarcomas—7<br>Rhabdomyosarcomas—6<br>Papilloma and sebaceous cyst—1<br>Sebaceous cyst leading to malignancy—1<br>Papilloma, sebaceous cyst and early fibrosarcoma—1<br>Sebaceous cyst and fibrosarcoma—4<br>Papilloma and fibrosarcoma—1<br>Fibrosarcoma and squamous carcinoma—1<br>Hidradenoma and fibrosarcoma—1 |

tumours originated a little distance away from it. He felt that the cells in the vicinity of the pellet, while failing in their attempt to isolate it by formation of a collagenous capsule probably due to toxic effect of the carcinogen, acquire greater reproductive activity until they reach the phase of rapid, uncontrolled growth. Throughout this study dermal collagen in the skin adjacent to tumour masses was thickened, hyalinised and tinctorially altered (Bhisey, 1965). Vasiliev and Guelstein (1963) infer that, compared to cells seen in earlier lesions, presarcomatous cells have an increased resistance to the toxic action of carcinogenic hydrocarbons. Hence they believe that alteration in cell sensitivity is an important factor in neoplastic transformation.

Many workers have observed that polycyclic hydrocarbons are taken up into the cytoplasm of cells (Richter and Saini, 1960 ; Sloane and Loeser, 1963), and have studied the relationship between cell damage produced by carcinogens and the process of tumour development. Recent cytochemical and fluorescent studies by Allison and Mallucii (1964) have shown that the carcinogenic hydrocarbons are concentrated in lysosomes. They believe that high concentrations of carcinogens in lysosomes could bring about considerable release of lysosomal enzymes into the cytoplasm. The presence of the freed hydrolases would damage the mechanism controlling cell division without impairing the mitotic capacity. They also attribute cytotoxicity of carcinogenic hydrocarbons to this release of lysosomal enzymes.

Fluorescent studies of carcinogens in skin by Simpson and Cramer (1943) have shown that immediately after a single application of carcinogen, the bulk of it is seen at two sites—in the sebaceous glands and the sebum and in the free lipids of the keratinized epithelium. Subsequently the sebaceous glands degenerate releasing sebum containing methylcholanthrene into the hair follicles and then on to the keratinized surface layer of the epidermis. According to these authors, in the later stages some specimens of skin showed fine fluorescent globules in circumscribed areas of the epidermis where small lipid globules are formed as a result of degeneration.

An important influence on the induction of skin cancer by subcutaneous injection of the carcinogen 20-methylcholanthrene appears to be the different pathways by which the carcinogen exercises its effect and the variable nature of the target cell. As to why a particular cell type should become malignant in response to its proximity to methylcholanthrene is probably dependent upon how susceptible it is at the moment to the altered tissue environment.

### SUMMARY

Fifty-eight Swiss albino mice were injected 0·5 mg. of 20-methylcholanthrene dissolved in thiophene free benzene. Twenty-four animals were killed at predetermined intervals of time in order to study early neoplastic changes in fibroblasts. Of the remaining 34 animals, 31 developed tumours. Histological data obtained from these growths emphasise that, in this strain of mouse, neoplasms occur following injection of carcinogen in the skin, but only locally. While squamous cancer predominates in the epidermis all dermal cell types respond to injected MCA with the presence of different tumour types of varied oncogenesis. The mode of administration of the carcinogen decides how multiple its target action is at the cell level.

### REFERENCES

Allison, A. C. and Mallucii, L.—(1964) *Nature, Lond.*, **203**, 1024.
Bhisey, R. A.—(1965) ' Biological studies with the electron microscope with reference to the fibroblast in experimental hormonal stress and induced neoplasia '. M.Sc. Thesis, Bombay University.
Bonser, G. M.—(1940) *Am. J. Cancer*, **38**, 319.
Burdette, W. J. and Strong, L. C.—(1943) *Cancer Res.*, **3**, 13.
Burrows, H., Hieger, I. and Kennaway, E. L.—(1932) *Am. J. Cancer*, **16**, 57.
Haddow, A.—(1935) *Nature, Lond.*, **136**, 868.
Orr, J. W.—(1939) *J. Path. Bact.*, **46**, 495.
Richter, K. M. and Saini, V. K. —(1960) *Am. J. Anat.*, **107**, 209.
Shimkin, M. B. and Andervont, H. B.—(1940) *J. natn. Cancer Inst.*, **1**, 57.
Simpson, W. L. and Cramer, W.—(1943) *Cancer Res.*, **3**, 362.
Sloane, G. H. I. and Loeser, C. M.—(1963) *Cancer Res.*, **23**, 1555.
Strong, L. C.—(1941) *Cancer Res.*, **1**, 572.
Vasiliev, J. M. and Guelstein, V. I.—(1963) *J. natn. Cancer Inst.*, **31**, 1123.